# Reflecting on an incredible journey with JBC

**DOI:** 10.1016/j.jbc.2021.100875

**Published:** 2021-07-02

**Authors:** Lila M. Gierasch

Five years ago, I described the exciting circumstances that encouraged me to take on the role of Editor-in-Chief of the *Journal of Biological Chemistry* (JBC): the new opportunities offered by the shifting landscape of scientific publishing, the stunning progress happening across science but particularly in those areas central to JBC, and the enduring foundation of the journal itself, built on over a century of thoughtful and respectful stewardship (1). Nonetheless, by the measures that are generally used to assess the performance of a journal, JBC was in decline. Yet, I was convinced that there should be a bright future for JBC if the right actions were taken. My reasoning was simple: JBC has “the right stuff” for a journal. JBC is by scientists and for scientists. It exists to serve the community of biological chemists and help advance their science. The editorial team holds content to a very high standard of rigor and reliability, without being swayed by perceptions of scientific “sexiness.” JBC has provided a home for the significant findings of countless authors, enabling future generations of researchers to see farther and dig deeper. All that remained was to figure out what was leading to the disconnect between JBC’s metrics and the obvious respect and love for JBC on the part of a large fraction of the community of biological chemists and fix it. Simple, right? Understanding this dilemma fueled the incredibly busy last 5 years and catalyzed myriad innovations and adjustments in the JBC world. As I reflect on my time as the Editor-in-Chief, rapidly coming to an end, I’m pleased, optimistic, and proud of all the JBC family who worked so hard to reinvigorate our journal. Let me briefly touch on what I now think are the keys to a healthy, vibrant JBC.

***Care about science*****.** I am so thrilled that we have changed our model to publish JBC’s content open access (2). This move removes all barriers to the content of JBC, period. Hats off to the American Society for Biochemistry and Molecular Biology (ASBMB) for making this huge step possible—while conceptually, the decision was easy, the logistics and finances were difficult to grapple with. Moving to open access in and of itself would not have a substantial impact if readers do not value and trust our content. Specifically, without its unimpeachable reputation for rigor and reproducibility, JBC might devolve into just another journal. During my term, we strengthened this reputation further, expanding our staff to check all papers for data integrity issues prior to publication (3). We published articles (https://www.asbmb.org/asbmb-today/authors/kaoru-sakabe), held webinars (https://youtu.be/Dduwng8GFwc), updated data availability policies (4), and used social media to raise awareness of best practices as well as the critical choices researchers make in data acquisition that avoid problems down the road. We also made the review process at JBC more transparent by publishing associate editor names on the articles they handled and by modifications to our review forms (5).

***Care about the authors and readers*****.** Early on in my tenure, we discovered that there had been a kind of “if we build it, they will come” attitude at JBC—that readers should intuitively understand that JBC content is great and come to us. Instead, we realized that we needed to deliver content to our readers, serving them and the authors of these studies. We embraced social media (most recently illustrated by our annual “Methods Madness” tournament!; https://www.jbc.org/methods_madness_2021), we created curated emails to share the exciting papers in JBC to a broad audience, we launched the Editors’ Pick Highlights articles to bring attention to key findings published in JBC, and we began a program of editing titles and abstracts to enhance their readability and accessibility (6). We also improved the process for authors: formalizing an appeal committee, clarifying reviewer expectations, and continually working to decrease the time from submission to first decision (always fast, and now a remarkable 16 days). One major endeavor was the launching of JBC Reviews to replace Mini-Reviews (7). We heard from authors that they wanted more flexibility than Mini-Reviews allowed in order to capture the excitement in their fields, and we heard from readers and other educators that they wanted fantastic imagery. We have invested a huge amount of editorial, staff, and artistic resources to bring JBC Reviews to life, and the resulting articles are a terrific mix of instructional guides and resources, provocative proposals, paradigm shifts, and much more.

***Care about the authors-to-be.*** Many scientists who were doing science back in the 20th century are well aware of JBC’s important role in the scientific publishing landscape, but younger generations have grown up in an era where many new journals have been and continue to be launched, creating a maze to navigate in order to connect with JBC. To break down some of those obstacles and help advance the careers of these researchers, we have created an Early Career Reviewer board (8), redesigned our Editors’ Picks papers to better feature first authors, and reimagined our Herbert Tabor Award program to honor junior authors of top content in JBC, including recognition at the ASBMB annual meeting (9). Moreover, we have created a committee on inclusion, diversity, and engagement, jointly with the other two ASBMB journals. This group has only recently begun their work, but the energy and deep commitment to inclusivity, finding solutions to bias, and enhancing the engagement of all in the publishing process for ASBMB journals is palpable.

***Care about the reviewers*****.** JBC’s associate editors and editorial board members play critical roles in evaluating papers, contributing their own best work, and charting the course of JBC’s future (8, 10). They exemplify the fact that scientists are the ultimate owners of the journal. During my tenure, we have strengthened the bonds among all the editorial leaders of JBC—expanding training and resources, channeling their energy into special projects, rebuilding lines of communication, and spending more time listening than ever before.

***Care about the staff*****.** While the editors play a visible role as the scientific stewards of the journal, the ASBMB staff plays an often silent but no less critical role in the success of our endeavor. Without their extensive knowledge, their ability to support every aspect of journal operations, their care and patience in working through complicated situations with editors and authors, their enthusiasm in highlighting our content, and their creativity in solving problems, there simply would not be a JBC.

The changes and innovations described above, along with many more small but important updates, could not have happened without the support of ASBMB and the contributions of the entire JBC team. What a wonderful group of colleagues! I thank all of you from the bottom of my heart—it has been a joy working with you!

So now, it only remains to pass the baton and encourage the next Editor-in-Chief to continue to enrich JBC. My term ends at the end of June. My successor has not been named as of this writing, but Fred Guengerich will take the reins as the interim Editor-in-Chief until the next editor is appointed. I am confident that Fred, the new Editor, and the entire team will continue to pilot this wonderful journal to a bright future. A toast to them, to our authors and readers, and to JBC!

## References

[1] Gierasch L.M. (2016). The Journal of Biological Chemistry: 2016 Onward. J. Biol. Chem..

[2] Gierasch L.M. (2021). Welcome to a new year and a new OPEN ACCESS JBC!. J. Biol. Chem..

[3] McCormick C.D., Sakabe K. (2019). Ensuring the quality of data published in JBC is a team effort.

[4] Gierasch L.M., Davidson N.O., Rye K.-A., Burlingame A.L. (2020). The data must be accessible to all. J. Biol. Chem..

[5] Guengerich F.P., Gierasch L.M. (2017). What happens when you submit a paper to JBC?. J. Biol. Chem..

[6] Gierasch L.M. (2017). JBC is on a mission to facilitate scientific discovery. J. Biol. Chem..

[7] Gierasch L.M. (2019). Introducing JBC reviews. J. Biol. Chem..

[8] Gierasch L.M. (2019). Celebrating and cultivating excellent peer review at JBC. J. Biol. Chem..

[9] Gierasch L.M., DeMartino G. (2017). The Herbert Tabor Best Paper Awards: Celebrating young authors who contribute top content to JBC. J. Biol. Chem..

[10] Gierasch J.M. (2020). In JBC we trust. J. Biol. Chem..

